# Hatchery and wild larval lake sturgeon experience effects of captivity on stress reactivity, behavior and predation risk

**DOI:** 10.1093/conphys/coac062

**Published:** 2022-10-07

**Authors:** Lydia Wassink, Belinda Huerta, Doug Larson, Weiming Li, Kim Scribner

**Affiliations:** Department of Integrative Biology, Michigan State University, 288 Farm Lane, East Lansing MI 48824, USA; Department of Fisheries and Wildlife, Michigan State University, 480 Wilson Road, East Lansing MI 48824, USA; Department of Fisheries and Wildlife, Michigan State University, 480 Wilson Road, East Lansing MI 48824, USA; Department of Fisheries and Wildlife, Michigan State University, 480 Wilson Road, East Lansing MI 48824, USA; Department of Integrative Biology, Michigan State University, 288 Farm Lane, East Lansing MI 48824, USA; Department of Fisheries and Wildlife, Michigan State University, 480 Wilson Road, East Lansing MI 48824, USA

**Keywords:** survival, physiology, early ontogeny, cortisol, Captive rearing

## Abstract

Reintroduction programs are important tools for wildlife conservation. However, captive rearing environments may lead to maladaptive behavior and physiological alterations that reduce survival probability after release. For captive rearing programs that raise individuals captured from the wild during early ontogeny for later release, there is a lack of information about when during ontogeny the detrimental effects of captive rearing may become evident. In this study we compared cortisol levels, predation rates and swimming behavior between hatchery-produced and wild-caught larval lake sturgeon (*Acipenser fulvescens*), a threatened fish species, at three times over 9 days. Cortisol levels did not indicate that hatchery-produced individuals were more stressed, but cortisol reactivity to an acute stressor disappeared for both hatchery-produced and wild-caught larvae after 9 days in the hatchery. Swimming activity levels decreased over time for hatchery-produced larvae but increased over time for wild-caught larvae, suggesting that behavioral trajectories may be programmed prior to the larval stage. Neither increasing nor decreasing activity levels was advantageous for survival, as predation rates increased over time in captivity for larvae from both treatments. Results suggest that physiological and behavioral phenotypes may not accurately predict survival for individuals released from reintroduction programs and that the captive environment may inhibit transition to the wild even if cortisol levels do not indicate high stress. Findings emphasize that even a short amount of time in captivity during early ontogeny can affect phenotypes of individuals captured from wild populations, which may impact the success of reintroduction programs.

## Introduction

Reintroduction programs, including captive breeding and rearing programs that release individuals to increase numerical abundance and persistence of wild populations, are important tools for wildlife conservation (Clark and Westrum, 1989). However, release of captive individuals can be counterproductive to conservation goals by reducing fitness of wild populations, as traits adaptive in captive environments may be maladaptive in the wild (McPhee, 2004). Studies that focus on conditions in fish hatcheries have demonstrated that, due to possible domestication selection, mean population fitness may decline proportionally with the amount of time spent in a captive environment (Lynch and O’Hely, 2001; Ford, 2002). Hatchery-produced fish often experience higher risk of predator-based mortality compared with wild fish, as a result of altered antipredator behavior (Berejikian, 1995; Stunz and Minello, 2001; Alvarez and Nicieza, 2003; Huntingford, 2004) as well as lower reproductive success (Berejikian *et al.,* 1997; Araki *et al.,* 2009).

While the multi-generational effects of captivity have been well documented in salmonids (e.g. Houde *et al.,* 2010), there is still a need to understand within-generational effects of captive rearing on individuals in captive rearing or headstarting programs, as well as in other species. Captive rearing methods used for wildlife reintroduction programs raise wild-caught individuals for release, in order to maximize survival during vulnerable early life stages while avoiding the multi-generational effects of captivity. However, stress induced by captivity may result in maladaptive behaviors that may reduce post-release survival, thus hindering the goals of conservation programs seeking to numerically expand wild populations (McDougall *et al.,* 2006; Berger-Tal *et al.,* 2016). To inform conservation efforts, it is essential to understand how and when during early ontogeny individual phenotypes are affected by captive environments, so that maladaptive effects of captivity can be reduced.

A primary concern with captive rearing is the potential to expose individuals to stress during important developmental periods. Stress experienced during early life stages can cause long-term alteration to hypothalamic–pituitary–interrenal (HPI) stress axis function, which can affect stress-related behaviors (Auperin and Geslin, 2008; Lukkes *et al.,* 2009; Turner *et al.,* 2010). Chronic stress (stressors experienced continuously) causes chronic elevation of cortisol levels, which can lead to inhibition of the negative feedback loop of the stress axis, and result in stress axis hyperactivity (Schreck *et al.,* 1997; Pariante and Lightman, 2008; Jeanneteau *et al.*, 2012). Stress axis hyperactivity is characterized by significantly elevated cortisol levels. For example, chronically stressed zebrafish exhibited whole-body cortisol levels over twice as high as those of control zebrafish, as well as altered behavior (Piato *et al.,* 2011). Features of the captive environment, such as high density in fish hatcheries, has been shown to increase cortisol levels in individuals, indicating increased stress (Falahatkar *et al.,* 2009; Li *et al.,* 2012). In captive rearing programs, behavior has been shown to be important for predator avoidance, dispersal, foraging and reproduction (Harvey *et al.,* 2002; Kreger *et al.,* 2006; Okuyama *et al.,* 2010), but chronic stress inhibits development of these important behaviors and negatively affects transition to the wild and survival post-release (Olla *et al.,* 1998; Evans *et al.,* 2014). Therefore, even if captive-reared individuals were produced from wild parents, and thus are genetically adapted to the wild environment, stress experienced during early life stages in captivity can have profound behavioral effects that result in maladaptation when subsequently introduced to the wild.

Lake sturgeon (*Acipenser fulvescens*) are a regionally threatened species and are increasingly reared in hatchery conservation programs. Lake sturgeon conservation also is of cultural value for First Nations people in the Great Lakes region (LRBOI (Little River Band of Ottawa Indians), 2008; Mann *et al.,* 2011). Populations have been dramatically reduced in abundance and in distribution relative to historic levels because of over-exploitation and habitat disturbance (Ferguson and Duckworth, 1997). Populations remain vulnerable to environmental stressors associated with climate change and other anthropogenic threats (Hayhoe *et al.,* 2010; Comte *et al.,* 2013), leading to a need for hatchery conservation programs. Currently, lake sturgeon are a management priority in the Great Lakes (Hayes and Caroffino, 2012). Streamside rearing facilities raise larval lake sturgeon for release into local populations (Brown and Day, 2002; Holtgren *et al.,* 2007). In lake sturgeon, the stress axis is functional by the time individuals reach the larval stage and begin exogenous feeding, showing a physiological response to acute stress evidenced by an increase in cortisol levels (Zubair *et al.,* 2012; Earhart *et al.,* 2020b). Early life stress due to factors associated with captive rearing environments, such as high density, has been shown to increase cortisol levels in fish, indicating higher stress (Falahatkar *et al.,* 2009; Li *et al.,* 2012; Earhart *et al.,* 2020a). Features of hatchery rearing have been shown to cause stress for lake sturgeon, such as incubating eggs in McDonald jars (Earhart *et al*., 2020a) and rearing larvae at high densities after hatch (Bauman *et al.,* 2015). Early life stress induced by hatchery rearing may impact survival, since early life stress influences antipredator behaviors in larval lake sturgeon (Biro *et al.,* 2003; Wassink *et al.,* 2019). Much of the high mortality lake sturgeon experience during the first year of life is caused by predation during the early larval stage (Waraniak *et al.,* 2018). Therefore, the impact of hatchery stress on lake sturgeon larval behavior and predation has important conservation implications.

Our study compared hatchery-reared and wild-caught sturgeon larvae from the Black Lake system during the early larval stage to investigate whether short durations in captivity may detectably stress individuals. Stress during early ontogeny is especially likely to alter behavioral and physiological developmental trajectories, and thus husbandry practices during early stages are of interest to captive rearing programs. Our research objectives were to (i) determine whether the hatchery environment affects stress and stress reactivity in larval lake sturgeon, (ii) investigate whether rearing environment influences predation rates and ability to learn predator avoidance and (iii) determine whether wild-caught lake sturgeon experience hatchery-induced stress after spending time in the hatchery environment. We hypothesized that the hatchery rearing environment induces behavioral and physiological phenotypic changes via alteration to HPI axis functioning in larval sturgeon. Our predictions were that (i) hatchery-produced larvae would have higher cortisol levels and higher activity levels compared with wild-caught larvae, as these are indicators of chronic stress; (ii) hatchery-produced larvae would have higher predation rates compared with wild-caught larvae, as an indicator of maladaptation to the wild environment; and (iii) wild-caught larvae would more closely resemble hatchery-produced sturgeon in terms of cortisol levels, behavioral phenotype and predation rates after spending time in the hatchery.

## Materials and Methods

To investigate how early rearing environments shaped behavior, stress and survival in lake sturgeon, the experiment was conducted in May 2018 using lake sturgeon larvae produced at the Black Lake Sturgeon Facility and wild larvae collected from the Upper Black River during the period of larval dispersal. The Black Lake system enables a direct comparison between early rearing environments, specifically egg incubation and free embryo rearing in natural stream vs. hatchery conditions, for individuals produced from the same wild population.

Data collection was initiated ~8 days post-hatch for both hatchery-produced and wild-caught groups. For hatchery-produced larvae, eggs were collected from three female lake sturgeon and sperm was collected from three male lake sturgeon spawning in the Upper Black River in Onaway, MI, USA. Full-sibling offspring were produced using one-to-one crosses using standard lake sturgeon culture procedures (Crossman *et al.,* 2011; Bauman *et al.,* 2016). Fertilized eggs were incubated in McDonald jars, with 5 ml of eggs (approximately 260 eggs) per jar, supplied with flowing stream water at a rate of 56.78 l/hour until hatch. McDonald jars and flow were inspected daily to ensure movement of the 5 ml of eggs in each jar was maintained at a slow roll throughout the incubation period. After hatch, free embryos were moved to 3-liter aquaria supplied with flowing stream water at a rate of 56.78 l/hour and provided with 2.54 cm^3^ sinking Bioballs (*N* = 32; CBB1-S, Pentair AES) as artificial substrate until reaching the larval stage. For wild-caught larvae, individuals dispersing downstream from spawning areas at night were collected using 1000-micron D-frame drift nets and transported to the hatchery in a cooler supplied with aerated stream water. At the hatchery, wild-caught larvae were placed in 3-liter aquaria supplied with flowing stream water at a rate of 56.78 l/hour. No Bioballs were provided since wild-caught individuals had reached the larval stage.

We used equal numbers of hatchery-produced and wild-caught larvae. Hatchery-produced larvae were represented by three families, with six replicates within each family. Wild-caught larvae represented three nights of drift net sampling (rather than families), with six replicates within the group from each night. Each replicate contained 132 larvae for a total of 6336 larvae in the entire experiment. To avoid overcrowding, larvae from each replicate were divided among three 3-l aquaria, with a maximum density of 44 larvae per 3-l aquarium. Water was pumped directly from the river upstream of the hatchery into and through 100 micron and 50 micron sock filters and into a head tank and then distributed via gravity throughout the hatchery, including the experimental tanks used for the experiment. In the larval rearing area, water was subsequently filtered through 50 and 25 micron filters for additional solids removal. Each 3-l aquarium was supplied with filtered stream water at ambient stream temperature flowing at a rate of 56.8 l/hour. Both hatchery-produced and wild-caught larvae were fed premium-grade brine shrimp (*Artemia sp.,* BSEP16Z, Brine Shrimp Direct) four times a day as per Bauman *et al.* (2016). In lake sturgeon, cortisol levels during exogenous feeding are crucial for transition to exogenous food (Earhart *et al*., 2020b). Hatchery-produced larvae were fed beginning at the onset of exogenous feeding (~10 days after hatch), and wild-caught larvae were fed beginning on their first day in the hatchery. Subsequently, throughout the duration of the experiment larvae were fed at 3:00 am, 9:00 am, 4:00 pm and 8:00 pm daily for both treatments.

Trials were repeated three times over a total duration of 9 days after wild-caught individuals were brought to the hatchery. The goals of this experiment were to determine whether behavioral and physiological phenotypes of wild-caught lake sturgeon larvae would change over the 9-day period of captivity to phenotypes of hatchery-reared larvae. The first 9 days of the larval stage encompassed the period of larval dispersal in the wild (Duong *et al.,* 2011), during which larvae initiate exogenous feeding and drift downstream from spawning locations. Stress-related behavior is particularly important during this stage due to the high rate of predation larvae encounter during dispersal (Waraniak *et al.,* 2018). Cortisol sampling, measurements, behavior trials and predation trials were conducted at three sequential time periods during early larval development (hereafter referred to as stages): stage A (Day 1 of the larval stage for hatchery-produced larvae, or the day after capture for wild-caught larvae), stage B (Day 5 of the larval stage) and stage C (Day 9 of the larval stage). Stage A, the beginning of the larval stage, was defined for hatchery-produced larvae based on emergence from the substrate (leaving the Bioballs). Individuals captured from the stream were assumed to be at the beginning of the larval stage, since the drift period begins upon after free embryo yolk reserves have been deleted and emergence from the substrate. Wild-caught larvae were carefully inspected to determine that none had remaining yolk sac, in order to ensure to the extent possible that wild-caught larvae were at an age comparable with hatchery-produced individuals. No individuals were used more than once for samples or trials.

### Analyses of larval body size

Total body length was quantified for each of six larvae per replicate at each of the three stages (*n* = 648) using ImageJ software (National Institutes of Health, Bethesda, MD, USA, http://rsbweb.nih.gov/ij/). Photos of larvae were taken using a digital camera, including a ruler for body size calibration and measured to the nearest millimeter.

### Analyses of larval cortisol levels

Cortisol levels were quantified to determine whether the hatchery environment induces stress in larval lake sturgeon. Whole-body cortisol levels were quantified for larvae from each replicate at stages A, B and C. At each stage, samples were taken at baseline (meaning no acute stressor was applied), or 30 minutes after individuals were exposed to an acute stressor to document levels of cortisol elevation as a physiological response to the stressor. All cortisol samples were taken at approximately noon for consistency in diurnal cortisol fluctuations. Each cortisol sample contained six individuals. For baseline samples, six individuals were removed from each replicate aquarium in the experiment and immediately euthanized using an overdose of MS-222 according to approved Animal Use and Care protocols. For post-stress samples, the acute stressor was the novel environment behavioral trial, which involved placing six larvae into a 15.24-cm-diameter petri dish to record swimming activity for 4 minutes, and has been shown to be a stressor for larval sturgeon (Wassink *et al.,* 2019). After the 4-minute acute stressor, individuals were placed into a 3-l aquarium and allowed a 30-minute rest period before being euthanized. All samples (*n* = 72 per stage, or 216 samples overall) were stored at −80°C until cortisol analysis. For cortisol extraction, samples were homogenized and ethyl acetate was used as a solvent for liquid–liquid extraction. The organic layer was extracted and evaporated prior to reconstitution in methanol. HPLC-MS/MS analysis was conducted using a Waters Xevo TQ-S mass spectrometer (Waters, Millford, MA, USA) (Bussy *et al.,* 2017).

### Analyses of larval behavior

Three types of behavior trials were conducted to measure larval behavioral responses to different stimuli:

1) Novel environment trials: Larval swimming activity in the petri dish was recorded with no additional stimulus. Encountering the novel environment of the petri dish has been shown to be a stressor for sturgeon larvae (Wassink *et al.,* 2019).
2) Thump trials: Larval swimming activity in the petri dish was recorded after a 212 g weight was dropped onto the table surface from a height of 22 cm to induce a startle response.
3) Odor trials: Larval sturgeon have an innate antipredator response to alarm cues released from the skin of injured conspecifics that facilitates learning of predator odors (Wishingrad *et al.,* 2014; Sloychuk *et al.,* 2016), an ecologically important cognitive function. Larval swimming activity in the petri dish was recorded after larvae were exposed to odor created from whole-body homogenization of sacrificed sturgeon larvae, as alarm cues in tissue homogenate from conspecifics has been shown to cause a physiological and behavioral stress response in sturgeon (Wishingrad *et al.,* 2014; Bjornson *et al.,* 2020). At the start of the trial, 1 ml of odor was added to the center of the petri dish using a pipette.

One of each type of behavioral trial was conducted using six individuals from each of six replicates in each treatment (hatchery and wild). Trials took place in a 6-inch petri dish and behaviors of all individuals were recorded using a Go-Pro Hero 4 camera (GoPro, Inc.) for a duration of 4 minutes. Petri dishes were supplied with the same water used in aquaria, so larvae did not experience a change in water quality, temperature or dissolved oxygen. The volume of water in each petri dish was ~0.36 l. Behavior trials employing the same acute stimuli were conducted for individuals at each of the three stages (A, B and C) (*n* = 648 individuals per stage, or 1944 total).

Loligo® software was used to simultaneously track activity of the six individuals in each replicated trial, following Sakamoto *et al.* (2016). A center zone was defined that excluded a 1-inch perimeter around the petri dish edge to quantify edge-seeking behavior. Variables quantified from the entire 4-minute video period included each individual’s velocity (cm/s), acceleration (cm/s^2^), percent time active, total distance traveled (cm), number of visits to center zone and time (s) spent in center zone (Wassink *et al.,* 2019; Wassink *et al.,* 2020).

### Predation

Predation rates were quantified using rusty crayfish (*Orconectes rusticus*), an important predator of larval lake sturgeon (Crossman *et al*., 2018). Trials were designed following Wassink *et al.,* 2019, which demonstrated significant differences in crayfish predation of larval sturgeon from different stress treatments (Wassink *et al.,* 2019). One set of predation trials was conducted using lake sturgeon larvae naïve to crayfish, and a second set of trials was conducted using larvae conditioned to crayfish odor combined with dead conspecific alarm cues, in order to observe whether rearing environment affected the ability to learn predator odor. Larvae were removed to a separate 3-l tank for conditioning to predator odor. Conditioning odor was created by combining crayfish odor (30 ml of water from a 15.24 × 22.86 cm container housing three crayfish for 1 hour) and lake sturgeon conspecific alarm cue (20 ml of water containing sturgeon larvae homogenate). Sturgeon larvae homogenate was created from whole-body homogenization of sacrificed sturgeon larvae, using approximately 15 individuals of the same age and size as experimental larvae. A total of 4 ml of conditioning odor was added to each 3-l tank housing larvae the night before the predation trial, and an additional 4 ml of conditioning odor was added to each tank the following morning ~8 hours prior to the predation trial.

Predation trials were conducted at each of the three stages (A, B and C) and included a set of trials with larvae naïve to predator odor and a set of trials with larvae conditioned to predator odor combined with alarm cues. No larvae were used for more than one trial. Each trial at each stage used a unique individual crayfish, so that crayfish were not used for more than one trial. One naïve predation trial and one conditioned predation trial was conducted per replicate. Conditioned predation trials were conducted 24 hours after naïve predation trials at each stage.

Predation trials took place in tanks that measured 42 × 30 cm, with a water depth of 12 cm (volume, 15.12 l). Tanks were supplied with flowing stream water at a rate of 56.8 l/hour, at ambient stream temperature (mean daily temperature ± standard deviation = 17.1°C ± 1.07). Rusty crayfish (*O. rusticus*) were collected from the Upper Black River using minnow traps. Crayfish were captured ~24 hours before being used in a trial and maintained in tanks in the hatchery supplied with flow-through stream water. All crayfish used in the experiment were chosen to ensure approximate consistency in size based on carapace length (mean carapace length, 3.24 cm; standard deviation, 0.39 cm). No cover was provided in the tanks for larvae to hide from crayfish; however, tank size was chosen to give adequate room for larvae to actively avoid crayfish. A prior study by Crossman *et al*. (2018) demonstrated that larval lake sturgeon are more likely to position themselves in the water column than on substrate when in the presence of a crayfish predator, suggesting that larvae use vertical tank space to stay out of reach of crayfish and providing substrate as cover would not have aided predator avoidance. For each trial, 10 sturgeon larvae were placed into the bare (no substrate) tank and allowed to acclimate for 50 minutes. After acclimation, one crayfish was added to each tank and then removed after 2.5 hours. Surviving larvae were counted and removed from the tank (*n* = 72 trials per stage, or 216 trials total).

### Statistical analysis

Normality for body size and cortisol datasets was assessed using a Shapiro–Wilk test in R v 3.2.2. The body size and cortisol datasets were not normally distributed and therefore were log-transformed prior to analysis. Generalized Linear Models were fit using the *glm* function in R v 3.2.2. Models with delta AIC (Aikake Information Criterion)  < 2 were considered competitive for top model (Burnham and Anderson, 1998). The variables included in the body size models were treatment (hatchery-produced or wild-caught), stage (A, B and C) and the interaction of treatment and stage. The variables included in the cortisol models were treatment (hatchery-produced or wild-caught), stress state (baseline or post-stress) and the interaction of treatment and stress state. Predictor variables in the AICc selected models were further evaluated using ANOVA (Analysis of Variance).

For behavior datasets, dependent variables (percent activity, acceleration, velocity, distance, zone time and zone visits) were compressed into a composite behavioral measure using Principal Components Analysis (PCA). PCA was chosen following Ballew *et al.* (2017) as a means of handling inter-relatedness among measures of swimming behavior. Rather than considering closely related variables such as velocity, acceleration, etc., individually, the PCA is applied to reduce multiple dependent variables into fewer orthogonal components that can then be characterized as behaviors such as ‘boldness’ (e.g. Ballew *et al.,* 2017) depending on what aspect of behavior primarily informs each principal component. Thus, we used PCA to reduce dimensionality of the behavior dataset and facilitate interpretation of how independent variables affected measures of behavior.

The broken stick method was used to determine that PC1, PC2 and PC3 were significant (Jackson, 1993). Factor loadings above 0.5 were used to determine behavioral relevance of each principal component. Generalized Linear Models were selected for the three principal components using AICc model selection. ANOVA was used to conduct *F*-tests on the model output and determine which variables were significant (*P* < 0.05).

For the predation dataset, Generalized Linear Models using a negative binomial distribution were fit for the dataset of surviving larvae per tank using the *nb.glm* function in R v 3.2.2. Variables in the models included treatment (hatchery-produced or wild-caught), stage (A, B or C), conditioning treatment (naïve or conditioned to odor) and the two-way factor interactions. Variables present in the AICc selected model were further evaluated using a Chi-Square test.

### Animal welfare considerations

All experiments were conducted under approved Michigan State University Animal Use and Care protocols (04/17-071-00). Incidental stress was minimized for all lake sturgeon in the experiment to the extent possible. Captured adults were handled for only ~4 minutes each, and care was taken to ensure heads and gills remained underwater. In the hatchery, free embryos were provided with 2.54 cm^3^ sinking Bioballs (*N* = 32, CBB1-S, Pentair AES) as artificial substrate until emergence. At the onset of exogenous feeding at the beginning of the larval stage, larvae were supplied with food *ad libitum*. Tanks housing free embryos and larvae were cleaned daily, and mortalities were removed daily. Flow rate of filtered stream water was maintained at 56.8 l/hour to ensure adequate oxygenation. During the dispersal period or wild larvae, individuals were kept in a large cooler filled with stream water that was oxygenated using an aerator. Larvae used for behavior trials were supplied with oxygenated water and only left in the petri dish for the duration of the 4-minute trial. Any larvae sacrificed for samples or to create odor were euthanized using an overdose of MS-222 according to approved Animal Use and Care protocols.

## Results

### Larval body size

**Figure 1:**
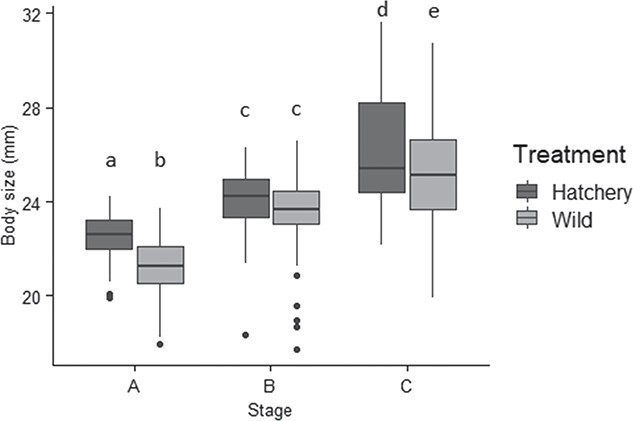
Body size (data on original scale, prior to log-transformation) across all three stages for both treatments: stage A (day one of the larval stage for hatchery-produced larvae, or the day after capture for wild-caught larvae), stage B (day five of the larval stage), and stage C (day nine of the larval stage) (n = 216 individuals per stage, or 648 individuals total). Size increased significantly across stages (p < 0.0001). Tukey HSD indicated that hatchery larvae were significantly larger than wild larvae at stages A and C, but not at stage B. Whiskers indicate minimum and maximum values, excluding data points (represented by dots) beyond 1.5 x the interquartile range for the upper and lower quartiles. Letters indicate results of Tukey HSD test.

The AICc selected model for body size included treatment, stage and the interaction of treatment and stage (Supplementary Table S1). ANOVA indicated that the main effect of body size was significant and the interaction of stage and treatment was significant (body size, *P* < 0.0001; stage*treatment, *P* = 0.028). In addition, the ANOVA and a post-hoc Tukey HSD indicated that hatchery-produced larvae were significantly larger than wild-caught larvae at stage A (Cohen’s D = 1.11; *P* < 0.0001) and at stage C (Cohen’s D = 0.48; *P* = 0.0001), but that there was no significant difference in size between treatments at stage B (*P* = 0.170) (Fig. 1).

**Table 1:** Factor loadings and eigenvalues for principal components analysis of behavioral variables

	Factor loadings						Eigenvalue	Variance %	Cumulative variance %
	*Velocity*	*Accel.*	*Zone time*	*% activity*	*Distance*	*Zone visits*			
PCI	0.5247	0.5396	−0.0907	−0.545	−0.2158	−0.2763	2.7399	45.6651	45.6652
PC2	0.3582	0.3198	−0.2607	0.2025	0.6642	0.4683	1.7293	28.8209	74.4861
PC3	−0.1005	−0.1043	−0.8485	0.1587	0.0612	−0.4797	1.1002	18.3367	93.8228
PC4	0.1121	0.0657	0.4502	0.2493	0.494	−0.6886	0.3342	5.5696	98.3924
PC5	0.2547	0.3232	0.3338	0.7543	−0.5105	0.0011	0.0912	1.5196	99.912
PC6	0.7133	−0.6978	−0.0048	0.0167	−0.0616	0.0083	0.0053	0.0879	100

### Larval cortisol levels

**Figure 2:**
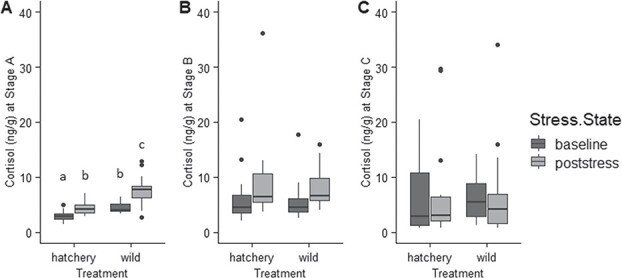
Body size (data on original scale, prior to log-transformation) across all three stages for both treatments: stage A (day one of the larval stage for hatchery-produced larvae, or the day after capture for wild-caught larvae), stage B (day five of the larval stage), and stage C (day nine of the larval stage) (n = 216 individuals per stage, or 648 individuals total). Size increased significantly across stages (p < 0.0001). Tukey HSD indicated that hatchery larvae were significantly larger than wild larvae at stages A and C, but not at stage B. Whiskers indicate minimum and maximum values, excluding data points (represented by dots) beyond 1.5 x the interquartile range for the upper and lower quartiles. Letters indicate results of Tukey HSD test.

For stage A (Day 1 of the larval stage), the AICc selected model included treatment and stress state (Supplementary Table S2). ANOVA indicated wild-caught larvae had significantly higher whole-body cortisol than hatchery-produced larvae at stage A (*P* < 0.0001, Cohen’s D = 1.26) (Fig. 2). Post-stress cortisol levels were also significantly higher than baseline cortisol levels at stage A for both treatments (*P* < 0.0001, Cohen’s D = 1.11) (Fig. 2). For stage B (Day 5 of the larval stage), the AICc selected model included stress state only (Supplementary Table S2). ANOVA indicated that post-stress cortisol levels were significantly higher than baseline (*P* = 0.0008, Cohen’s D = 0.83) (Fig. 2). For stage C (Day 9 of the larval stage), the AICc selected model was the null model (Supplementary Table S2), indicating that no variables were important in explaining variation in cortisol levels at this stage.

### Larval behavior

**Figure 3:**
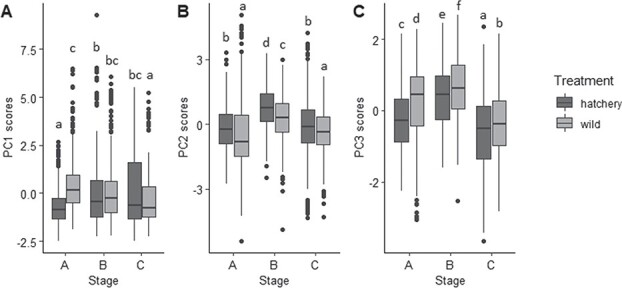
Principal Components for hatchery-produced and wild-caught larval behavior at each stage (n = 648 individuals per stage, or 1,944 individuals total). For PC1 (can be characterized as “inactivity”), hatchery-produced larvae had lower mean estimates of PC1 than did wild-caught larvae at all stages (averaged across trial type) (p < 0.0001). For PC2 (can be characterized as “total distance moved”), hatchery-produced larvae moved greater distance than wild-caught larvae at all stages (p < 0.0001). For PC3 (can be characterized as “zone avoidance”), wild-caught larvae exhibited significantly higher center zone avoidance compared to hatchery-reared larvae at all three stages (averaged across trial type) (p < 0.0001). Whiskers indicate minimum and maximum values, excluding data points (represented by dots) beyond 1.5 x the interquartile range for the upper and lower quartiles. Letters indicate results of Tukey HSD tests conducted for each Principal Component.

Behavioral traits associated with swimming activity (percent activity, velocity, acceleration, distance, zone time and zone visits) were reduced into three components using PCA. Factor loadings indicated that the most important variable contributing to variation along PC1 was percent activity (Table 1; Fig. 3; Supplementary Fig. S1). PC1, which explained 45.67% of the variation in the data, was negatively associated with percent activity and can be characterized as ‘inactivity’. The AICc selected model for PC1 included treatment, stage, trial and the interactions of treatment and trial (novel environment, thump or odor), trial and stage and treatment and stage (Supplementary Table S3). ANOVA indicated that all terms included in the model significantly affected PC1: treatment (*P* < 0.0001), stage (*P* = 0.0008), trial (*P* = 0.0040), treatment*trial (*P* = 0.0066), stage*trial (*P* = 0.0028) and treatment*stage (*P* < 0.0001). Hatchery-produced larvae had lower mean estimates of PC1 than did wild-caught larvae at all stages (averaged across trial type) (Cohen’s D = 0.17), indicating that hatchery-produced larvae were more active than wild-caught larvae regardless of developmental stage. At each stage, hatchery-produced larvae showed decreasing activity levels (indicated by an increase in mean PC1 scores), while wild-caught larvae increased their activity levels (indicated by a decrease in mean PC1 scores).

Factor loadings indicated that the most important variable contributing to variation along PC2 was distance moved (Table 1; Fig. 3; Supplementary Fig. S2). PC2, which explained 28.82% of the variation in the data, was positively associated with distance and can be characterized as ‘total distance moved’. The AICc selected model for PC2 included treatment, stage, trial, the interaction of treatment and trial and the interaction of trial and stage (Supplementary Table S3). ANOVA indicated that all terms included in the model significantly affected PC2: treatment (*P* < 0.0001), stage (*P* < 0.0001), trial (*P* = 0.6411), treatment*trial (*P* = 0.0398) and stage*trial (*P* < 0.0001). Hatchery-produced larvae moved greater distances (higher PC2 means) than wild-caught larvae at all stages (averaged across trial type) (Cohen’s D = 0.29).

Factor loadings indicated that the most important variable contributing to variation along PC3 was ‘zone time’, or time spent in the center zone of the petri dish (Table 1; Fig. 3; Supplementary Fig. S3). PC3, which explained 18.34% of the variation in the data, is negatively associated with zone time and can be characterized as ‘zone avoidance’ (Table 1). The AICc selected model for PC3 included treatment, stage, trial, the interaction between treatment and trial and the interaction between trial and stage (Supplementary Table S3). ANOVA indicated that all terms included in the model significantly affected PC3: treatment (*P* < 0.0001), stage (*P* < 0.0001), trial (*P* = 0.014), treatment*trial (*P* = 0.014) and stage*trial (*P* = 0.010). Wild-caught larvae exhibited significantly higher center zone avoidance, or more time spent along the 1-inch perimeter of the petri dish outside of the center zone, compared with hatchery-reared larvae at all three stages (averaged across trial type) (Cohen’s D = 0.28).

### Levels of larval predation

**Figure 4:**
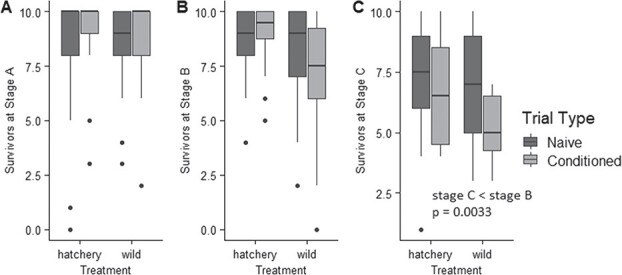
Survival of hatchery-produced and wild-caught larvae across three stages, including naïve to predator (first exposure) treatment and conditioned to predator odor (second exposure) treatment. Predation trials were conducted for each replicate, one trial using larvae that were naïve to predator odor and one using larvae that were conditioned to predator odor combined with alarm cues (n = 72 trials per stage, or 216 trials total). A Chi Square test indicated the effect of stage was significant (p < 0.0001) and the effect of treatment was not significant (p = 0.051). Pairwise comparisons indicated that survival was not significantly different from stage A to stage B (p = 0.365), but decreased significantly from stage B to stage C (p = 0.003). Whiskers indicate minimum and maximum values, excluding data points (represented by dots) beyond 1.5 x the interquartile range for the upper and lower quartiles.

For predation trials, the AICc selected model included stage and treatment (Supplementary Table S4). A Chi Square test indicated the effect of stage was significant and the effect of treatment was not significant (stage, *P* < 0.0001; treatment, *P* = 0.051). Pairwise comparisons indicated that survival was not significantly different from stage A to stage B (*P* = 0.365), but decreased significantly from stage B to stage C (*P* = 0.003) (Fig. 4).

## Discussion

Our findings demonstrated that stress-related phenotypes may develop over very short periods of time following the onset of captive rearing. Both hatchery-produced and wild-caught sturgeon experienced effects of the hatchery environment as indicated by physiological and behavioral differences among stages. Wild-caught larvae initially exhibited elevated physiological responses to an acute stressor as evidenced by higher whole-body cortisol (Fig. 2). Behaviorally, wild-caught larvae exhibited greater tendencies to maximize concealment (lower activity, lower total distance traveled and less time spent in the center of the petri dish). Wild-caught and hatchery-produced larvae exhibited no differences in survival during predation trials, and both members of groups experienced decreasing survival over time (Fig. 4) despite the advantage of increasing size (Fig. 1). Likewise, stage C larvae from both treatments showed no post-stress elevation of cortisol levels (Fig. 2). Wild-caught sturgeon, despite having spent early ontogenetic stages in the wild, were still affected by the captive rearing environment over a short period to the extent that their stress axis function was indistinguishable from that of hatchery-produced larvae after only 9 days.

One important caveat is that while age was estimated to be comparable with that of hatchery-produced larvae, exact fertilization and hatch dates were unknown for wild larvae. Small differences in age may have influenced cortisol levels, as cortisol levels change rapidly during development for lake sturgeon (Earhart *et al*. 2020b; Zubair *et al.,* 2012), and may also have influenced how each behaviorally responded to stressors experienced in the hatchery environment. Lake sturgeon developmental rate is highly influenced by temperature (Kempinger, 1988; Smith and King, 2005), which may have differed slightly between wild and hatchery environments despite the hatchery sourcing water from the natal stream. However, despite possible differences in developmental rates and hatch dates, both hatchery-produced and wild-caught larvae were at the onset of the larval drift period of the lake sturgeon life cycle, which is when physiological and behavioral stress-related responses to predators are particularly important (Waraniak *et al.,* 2018).

It is possible that the higher baseline cortisol levels observed in wild-caught larvae compared with hatchery-produced larvae at stage A may be a result of stress associated with capture and transitioning to a new food type. However, prior research supports the likelihood of hatchery environments affecting larval cortisol. A study by Earhart *et al*. (2020a) on cortisol in hatchery-produced lake sturgeon indicated that larvae incubated as eggs using a tumbling regime in McDonald jars, a standard hatchery practice, had lower cortisol levels and delayed onset of cortisol production than non-tumbled individuals. Additionally, the decrease in physiological reactivity to stress over time for both treatment groups in this study suggests that hatchery rearing impacts stress axis function. Cortisol elevation in response to an acute stressor decreased over time for larval lake sturgeon from both treatments and was no longer evident by stage C (Day 9 of the larval stage) (Fig. 2). A prior experiment with lake sturgeon larvae observed that a decrease in physiological reactivity was associated with chronic stress (Wassink *et al.,* 2019). Therefore, the lack of physiological reactivity to an acute stressor suggests that the hatchery environment may represent a source of chronic stress for both groups. While it has been proposed that lower physiological reactivity to threats may be adaptive in some contexts (Blas *et al.,* 2007), this experiment showed that predation rates increased over time coincident with a decrease in cortisol reactivity. Therefore, decreasing physiological reactivity to stress does not appear to improve fitness by helping lake sturgeon larvae avoid predation. Further research could investigate the role cortisol responses play in fitness by defining in what situations a lack of physiological reactivity is adaptive. Importantly, the stressor used in measuring cortisol reactivity to acute stress was not the predator itself, but rather the petri dish environment experienced during behavior trials. Therefore, it is possible larvae may have a physiological response to encountering crayfish that we did not observe in this study. Additional investigation into stressor-specific responses of lake sturgeon larvae to a variety of stimuli, beyond standardized acute stressors and including ecologically relevant threats such as real predator encounters, may be warranted.

Overall, hatchery-produced sturgeon were more active and moved greater distances (Fig. 3), while wild-caught sturgeon spent more time avoiding the center zone (Fig. 3). In addition, hatchery larvae showed decreasing activity levels over time, while wild-caught larvae showed increasing activity levels (Fig. 3). Increased activity levels have previously been shown to be associated with chronic stress for lake sturgeon larvae (Wassink *et al.,* 2019). Therefore, for wild-caught larvae, the large increase in baseline cortisol levels between stage B and C (Fig. 2) and the increasing activity levels over time (Fig. 3) suggest that hatchery-related stressors may be particularly impactful for larvae hatched in the wild environment. In addition, the opposite change in activity level over time in hatchery-reared and wild-caught sturgeon highlights the responsiveness of early life behavioral phenotypes to environments. Behaviors could be influenced by factors acting prior to the larval stage, such as genetic differences, maternal effects, egg incubation environment or free embryo experience prior to emergence from the substrate (Dammerman *et al.,* 2015). For example, prior work with lake sturgeon free embryos suggests that maternal effects are reflected in familial differences in yolk sac egg provisioning, metabolism and subsequent activity levels after hatch (Wassink *et al.,* 2019). Egg incubation environment has also been shown to influence development (Walquist *et al*., in review; Dammerman *et al.,* 2020), which could have downstream effects on stress-related behavior. Furthermore, behavioral phenotype may not be directly linked to physiological phenotype, since differences in physiological reactivity between treatments abated over time while behavioral differences remained. Collectively, findings indicate that further investigations into behavior programming mechanisms other than stress in the early life environment are warranted. It is also important to note that this study only observed swimming behavior in petri dishes, which does not approximate the natural environment. Thus, the differences we observed among treatments may have limited application to behavioral patterns that would be observed in the wild stream environment. However, similarly designed petri dish trials have been previously demonstrated to reveal behavioral outcomes of early life stressors (Wassink *et al.,* 2019; Wassink *et al.,* 2020), and thus findings from this study indicate that stress associated with rearing environments has behavioral effects. Findings suggests the necessity for further investigation into proximate causes of antipredator behaviors, especially to determine if there are proximate mechanisms programming behavioral responses to threat that function independently of the stress axis.

In contrast to a prior study using identical tanks and similarly sized crayfish and larvae in which higher activity levels and larger size were associated with increased survival of larval lake sturgeon during predation trials with crayfish (Wassink *et al.,* 2019), the current experiment found that predation rates increased over time (Fig. 4). Furthermore, in this study wild-caught larvae increased activity levels over time with no associated increase in survival. The increasing size experienced by both treatment groups over time was associated with decreased, rather than increased, survival during predation trials. A study by Crossman *et al*. (2018) investigated crayfish predation on larval sturgeon aged 8–16 weeks in an environment that more closely matched the stream environment, providing a larger space (2.81 m^3^ volume tanks) and substrate for larvae to use for hiding from predators. Here too, larger size was advantageous for avoiding predation by crayfish, indicating that body size is likely important for survival in the wild (Crossman *et al*., 2018). Thus, the negative effect of the captive environment on anti-predator abilities may override advantages that would typically promote survival for larval lake sturgeon in the wild.

Exposure to predator odor did not help lake sturgeon avoid predation by crayfish (Fig. 4). Results contrast behaviors of lake sturgeon larvae at later development stages observed by Wishingrad *et al.* (2014), which involved dramatically increasing activity levels in response to predator odor combined with conspecific homogenate. Lake sturgeon larvae associate predator odor with alarm cues released from the skin of conspecifics, to which they have an innate reaction (Wishingrad *et al.,* 2014). One possible explanation is that in this experiment, lake sturgeon larvae increased swimming activity as a stress response to conditioning odor, thereby depleting energy prior to predation trials. A second possible explanation is that larval lake sturgeon do not as readily learn the odor of a novel predator they did not co-evolve with, as rusty crayfish are an invasive species. A third possibility is that the hatchery environment canalized behavior for both hatchery-produced and wild-caught individuals in such a way that the ability to learn predator odor was not promoted. It has been suggested that the capacity for learning is cued by variability in the environment, since plasticity is usually adaptive in situations where individuals encounter new stimuli (Kotrschal and Taborsky, 2010; Mettke-Hofmann, 2014). If the hatchery setting creates a homogenous rearing environment through predictability of surroundings, food availability and lack of predator encounters, learning may be inhibited. Our findings do not necessarily suggest that hatchery-reared lake sturgeon are unable to learn predator avoidance via conditioning with alarm cues, as the learning process may depend on larvae’s developmental stage and may be context dependent. Bjornson *et al.* (2020) observed that lake sturgeon responses to alarm cue change during development, with later (pre-winter) stages showing greater reactivity to alarm cues. In addition, reactions to alarm cues were shown to depend on contexts such as foraging opportunity, since lake sturgeon larvae were less reactive to alarm cues in the presence of food (Bjornson *et al.,* 2020). Our findings highlight the importance of studies linking cognitive ecology to conservation, in order to develop a more complete understanding of what environmental factors promote or inhibit learning. Conservation programs would benefit from an understanding of how animal cognition is impacted by captive environments, as cognitive abilities are likely important for post-release survival (Teixeira *et al.,* 2007).

Inter-individual variation in cortisol concentrations and predation rates appears to increase by stage C, illustrating the importance of individuality in studying effects of captive rearing environments (Fig. 2; Fig. 4). This finding is consistent with research showing that captive environments increased inter-individual variation in oldfield mice (*Peromyscus polionotus subgriseus*) (McPhee, 2004). Personality (temperament) is important in studies on conservation in predicting success of captive breeding, captive rearing and reintroduction programs (McDougall *et al.,* 2006). For example, amphibians exhibit consistent individual differences in boldness, exploration and activity, all of which are important in both captive breeding success and survival after reintroduction (Kelleher *et al.,* 2018) in contexts such as predation, foraging and dispersal (Cote *et al.,* 2010). While pinpointing the causes of inter-individual variation in cortisol levels is beyond the scope of this study, it is possible that the increasing variation we observed represents the cumulative effect of individual experience. If an individual’s physiological phenotype is continuously informed by environment, it is possible that environmental influence on phenotype increases with age, resulting in phenotypic variability among individuals. Research investigating the mechanisms driving inter-individual differences in behavior could be applied to conservation efforts to predict or promote success in released individuals (Healy and Jones, 2002; Powell and Gartner, 2011; Greggor *et al.,* 2014; Ballew *et al.,* 2017).

Parentage of wild-caught larvae is unknown, but it is likely that genetic effects played a role in determining how individuals responded developmentally to rearing environment (Dammerman *et al.,* 2015; Dammerman *et al.,* 2016). There was likely higher genetic variation in wild-caught larvae than in hatchery-reared larvae, since hatchery-reared larvae were represented by three full-sib families while more adults contributed to wild larvae (Crossman *et al.,* 2011). Non-genetic maternal effects are also important for lake sturgeon, as prior research has suggested that both maternal and offspring experience are important in programming offspring stress axis function (Wassink *et al.,* 2020). Thus, additive genetic and maternal effects likely explain some of the inter-individual variation in behavior and physiology expressed during experimental trials. Genetic differences between hatchery-reared and wild-caught larvae could also explain behavioral differences.

Overall, this study illustrates that even captive rearing programs that use individuals captured from wild populations may impose a rearing environment that results in physiological and behavioral changes that can decrease survival after release. The cortisol stress response of wild-caught lake sturgeon larvae became similar to that of hatchery-produced larvae after only 9 days in the hatchery. Even though both groups showed decreasing physiological reactivity to stress over time spent in the hatchery, predation rates remained high, suggesting that physiological measures of stress and stress reactivity may not accurately predict success of individuals after release. Similarly, physiological phenotype may not predict behavioral responses to threats, as behavioral differences between hatchery-reared and wild-caught lake sturgeon did not appear to be linked to stress physiology.

Negative effects of captivity on predator avoidance may override advantages, such as larger size and higher activity levels, which would typically promote survival. Both hatchery-produced and wild-caught lake sturgeon larvae experienced increasing predation mortality over time, with larger size and higher activity levels failing to convey an advantage for predator avoidance as expected from prior studies (Wassink *et al.,* 2019; Crossman *et al.,* 2018). Therefore, even behaviors not directly induced by stress (or, conversely, not directly related to welfare) are of concern for reintroduction programs. This finding suggests that reintroduction programs could utilize research in cognitive ecology to create environments that behaviorally and cognitively prepare individuals for facing challenges in the wild (Greggor *et al.,* 2014). While re-creating features of the wild environment within captive environments may not be logistically feasible, programs could instead focus on promoting learning, behavioral plasticity and adaptation to novelty, to prepare individuals to navigate the transition to the wild and the high variability in environments that will be encountered (Teixeira *et al.,* 2007; Kotrschal and Taborsky, 2010; Mettke-Hofmann, 2014). Implementing this strategy would require a more detailed understanding of which specific features of early rearing environments promote learning and resilience to environmental variability, as well as the developmental mechanisms involved. While this study focused on stress hormones, behavioral reactivity and predation rates, negative effects of the captive environment are likely to be expressed differently in different species. Therefore, future work investigating the effects of captivity on wildlife in reintroduction programs should focus on species-specific, ecologically relevant consequences for fitness.

## Funding

This work was supported by the Michigan Department of Natural Resources [APP 144032]; the Great Lakes Fishery Trust; the U.S. Fish and Wildlife Service Coastal Program [APP 135058]; Michigan State University AgBioResearch; and the Schrems West Michigan Trout Unlimited Graduate Fellowship.

## Conflicts of Interest

None are declared.

## Data Availability Statement

The datasets generated during the current study are available from the corresponding author upon reasonable request.

## Supplementary Material

Web_Material_coac062
