# Supplementary material for: Hatchery and wild larval lake sturgeon experience effects of captivity on stress reactivity, behavior and predation risk
Source: Conserv Physiol. 2022 Oct 7;10(1):coac062. doi: 10.1093/conphys/coac062 (PMC9547518; doi:10.1093/conphys/coac062)
Supplement: Web_Material_coac062 [file web_material_coac062.zip › July 2022 supplementary materials.docx]

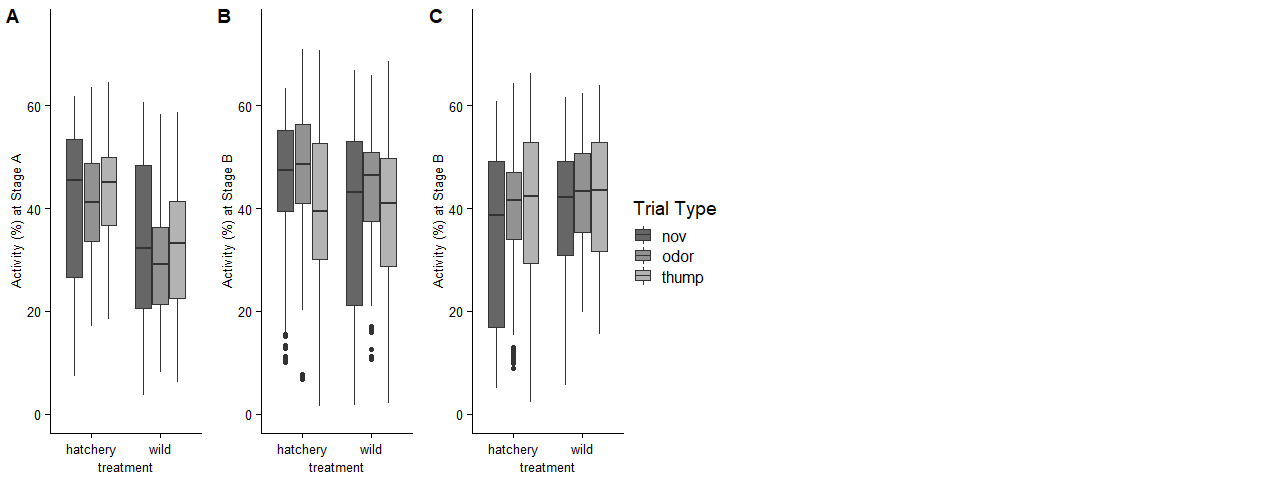


Supplemental Fig. S1: Percent activity for hatchery and wild larvae at all three stages (A, B, and C), for all three behavior trial types. Percent activity was the most important factor informing PC1. Whiskers indicate minimum and maximum values, excluding data points (represented by dots) beyond 1.5 x the interquartile range for the upper and lower quartiles.


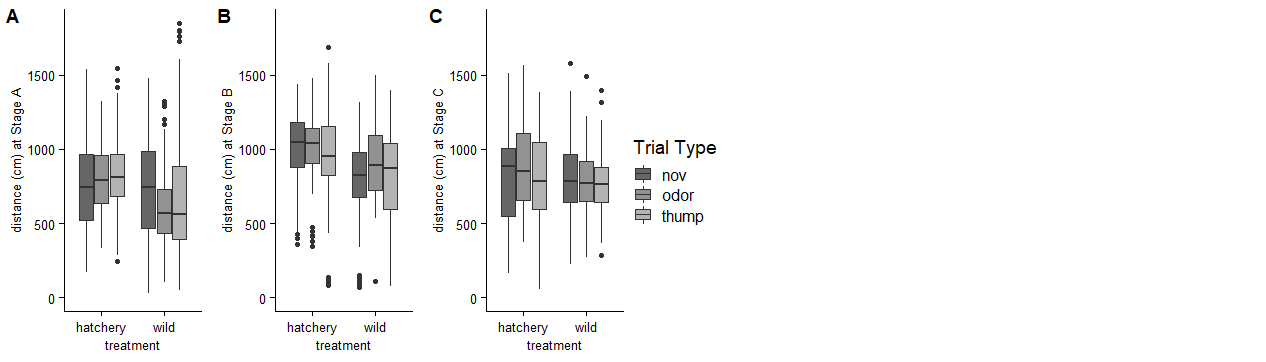


Supplemental Fig. S2: Total distance traveled (cm) during a four-minute behavior trial for hatchery and wild larvae at all three stages (A, B, and C), for all three behavior trial types. Distance was the most important factor informing PC2. Whiskers indicate minimum and maximum values, excluding data points (represented by dots) beyond 1.5 x the interquartile range for the upper and lower quartiles.


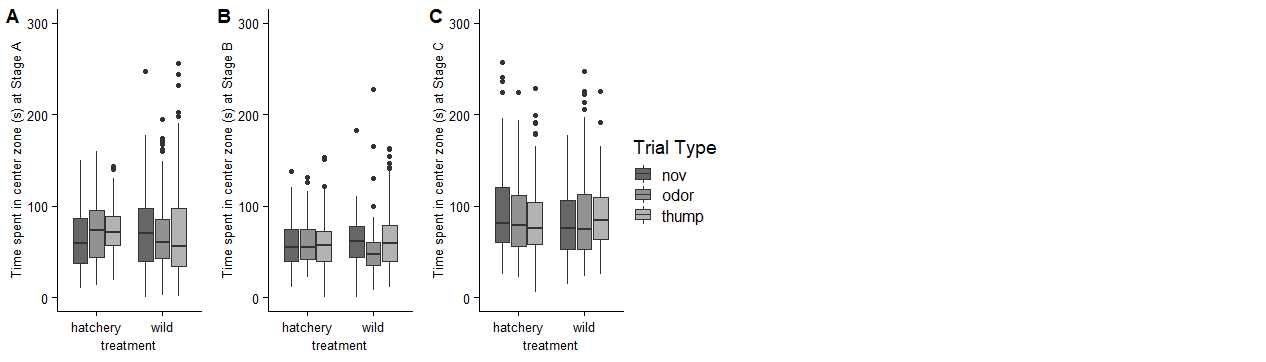


Supplemental Fig. S3: Time spent in center zone (s) for hatchery and wild larvae at all three stages (A, B, and C), for all three behavior trial types. The center zone was defined by excluding a one-inch perimeter around the edge of the petri dish. Zone time was the most important factor informing PC3. Whiskers indicate minimum and maximum values, excluding data points (represented by dots) beyond 1.5 x the interquartile range for the upper and lower quartiles.

| Supplemental Table 1: AICc selected models for body size, including predictor variables of treatment (hatchery or wild) and stage (A, B, or C) | | | |
| --- | --- | --- | --- |
| **Model** | **AICc** | **Delta AICc** | **Weight** |
| Length ~ Treatment + Stage + Treatment*Stage | -2727.8 | 0 | 0.83 |
| Length ~ Treatment + Stage | -2724.67 | 3.13 | 0.17 |
| Length ~ Treatment | -2672.54 | 55.26 | 0 |
| Length ~ Stage | -2309.88 | 417.92 | 0 |
| null model | -2282.63 | 445.17 | 0 |

| Supplemental Table 2: AICc selected models for whole-body cortisol levels at each stage (A, B, and C), using log-transformed cortisol dataset, including predictor variables of treatment (hatchery or wild) and stress state (baseline or post-acute stressor) | | | | | | |
| --- | --- | --- | --- | --- | --- | --- |
| **Model** | | **AICc** | **Delta AICc** | | **Weight** | |
| *Stage A* | | | | | | |
| Cortisol ~ Treatment + Stress State | | -81.36 | 0 | | 0.73 | |
| Cortisol ~ Treatment + Stress State + Treatment*Stress State | | -79.38 | 1.98 | | 0.27 | |
| Cortisol ~ Treatment | | -53.03 | 28.33 | | 0 | |
| Cortisol ~ Stress State | | -48.3 | 33.06 | | 0 | |
| null model | | -30.92 | 50.44 | | 0 | |
| *Stage B* | | | | | | |
| Cortisol ~ Stress State | | -11.02 | 0 | | 0.69 | |
| Cortisol ~ Treatment + Stress State | | -8.86 | 2.17 | | 0.23 | |
| Cortisol ~ Treatment + Stress State + Treatment*Stress State | | -6.57 | 4.46 | | 0.07 | |
| null model | | -1.55 | 9.48 | | 0.01 | |
| Cortisol ~ Treatment | | 0.57 | 11.59 | | 0 | |
| *Stage C* | | | | | | |
| null model | | 132.02 | 0 | | 0.42 | |
| Cortisol ~ Treatment | | 132.7 | 0.69 | | 0.3 | |
| Cortisol ~ Stress State | | 134.04 | 2.02 | | 0.15 | |
| Cortisol ~ Treatment + Stress State | | 134.8 | 2.78 | | 0.1 | |
| Cortisol ~ Treatment + Stress State + Treatment*Stress State | | 137.02 | 5.01 | | 0.03 | |
| Supplemental Table 3: AICc selected models for principal components associated with behavioral measurements, including only top four competitive models and null model, and predictor variables of treatment (hatchery or wild), stage (A, B, or C) and trial type (novel environment, odor, or thump) | | | | | | |
| **Model** | **AICc** | | | **Delta AICc** | | **Weight** |
| *PC1* | | | | | | |
| PC1 ~ treatment + stage + trial + treatment*trial + trial*stage + treatment*stage | 7136.86 | | | 0 | | 0.98 |
| PC1 ~ treatment + stage + trial + treatment*trial + treatment*stage | 7144.71 | | | 7.85 | | 0.02 |
| PC1 ~ treatment + stage + trial + treatment*stage | 7150.93 | | | 14.07 | | 0 |
| PC1 ~ treatment + stage + trial + treatment*trial + trial*stage | 7207.65 | | | 70.79 | | 0 |
| null model | 7248.5 | | | 111.63 | | 0 |
| *PC2* | | | | | | |
| PC2 ~ treatment + stage + trial + treatment*trial + trial*stage | 6180.05 | | | 0 | | 0.47 |
| PC2 ~ treatment + stage + trial + treatment*trial + trial*stage + treatment*stage | 6180.48 | | | 0.43 | | 0.38 |
| PC2 ~ treatment + stage + trial + trial*stage | 6182.39 | | | 2.34 | | 0.15 |
| PC2 ~ treatment + stage | 6206.65 | | | 26.59 | | 0 |
| null model | 6381.42 | | | 201.36 | | 0 |
| *PC3* | | | | | | |
| PC3 ~ treatment + stage + trial + treatment*trial + trial*stage | 5147.46 | | | 0 | | 0.54 |
| PC3 ~ treatment + stage + trial + treatment*trial + trial*stage + treatment*stage | 5148.42 | | | 0.97 | | 0.33 |
| PC3 ~ treatment + stage + trial + trial*stage | 5151.91 | | | 4.46 | | 0.06 |
| PC3 ~ treatment + stage + trial + treatment*trial | 5152.62 | | | 5.17 | | 0.04 |
| null model | 5529.48 | | | 382.02 | | 0 |

| Supplemental Table 4: AICc selected models for larval lake sturgeon predation data, including the top four competitive models and null model, and predictor variables of treatment (hatchery or wild), stage (A, B, or C) and carapace (crayfish carapace length as a proxy of crayfish size) | | | |
| --- | --- | --- | --- |
| **Model** | **AICc** | **Delta AICc** | **Weight** |
| Mortalities ~ Stage + Treatment | 710.50 | 0 | 0.45 |
| Mortalities ~ Stage | 712.15 | 1.65 | 0.20 |
| Mortalities ~ Treatment + Carapace + Stage | 712.27 | 1.77 | 0.19 |
| Mortalities ~ Stage + Exposure | 714.09 | 3.59 | 0.08 |
| null model | 726.93 | 16.43 | 0 |
